# The Impact of Financial Toxicity on Mental Health of LGBTQ+ Cancer Caregivers in the United States

**DOI:** 10.1002/pon.70639

**Published:** 2026-11-10

**Authors:** Kelly R. Tan, Camille R. Murray, Shaun R. Jones, Echo L. Warner, Madison Black, Sarah Belcher, Brittany M. Charlton, Lorinda A. Coombs, Heidi S. Donovan, Barbara L. Fredrickson, Erin E. Kent, Hazel B. Nichols, Hanyue Zhang, Austin R. Waters

**Affiliations:** ^1^ School of Nursing University of Pittsburgh Pittsburgh Pennsylvania USA; ^2^ UPMC Hillman Cancer Center Pittsburgh Pennsylvania USA; ^3^ Gillings School of Global Public Health University of North Carolina at Chapel Hill Chapel Hill North Carolina USA; ^4^ Lineberger Comprehensive Cancer Center University of North Carolina at Chapel Hill Chapel Hill North Carolina USA; ^5^ Cancer Control and Population Sciences Huntsman Cancer Institute Salt Lake City Utah USA; ^6^ College of Nursing Ohio State University Columbus Ohio USA; ^7^ Harvard T.H. Chan School of Public Health Boston Massachusetts USA; ^8^ Harvard Medical School and Harvard Pilgrim Health Care Institute Boston Massachusetts USA; ^9^ School of Nursing University of North Carolina at Chapel Hill Chapel Hill North Carolina USA; ^10^ Department of Psychology & Neuroscience University of North Carolina at Chapel Hill Chapel Hill North Carolina USA; ^11^ Division of Population Sciences Department of Medical Oncology Dana‐Farber Cancer Institute Boston Massachusetts USA

**Keywords:** cancer, caregiving, financial toxicity, mental health

## Abstract

**Introduction:**

Financial toxicity and poor mental health are common among lesbian, gay, bisexual, transgender, queer, and other non‐cisgender heterosexual (LGBTQ+) cancer caregivers. This study aimed to assess the relationship between financial toxicity and mental health among LGBTQ+cancer caregivers.

**Methods:**

We conducted a sequential explanatory mixed‐methods study (national survey and semi‐structured interviews with a subset of survey participants). Data were collected from April 2024 through December 2024. Bivariate and multivariable regression models were used to examine the association between financial toxicity (modified COmprehensive Score for financial Toxicity [mCOST]) and mental health outcomes (Mental Health Continuum‐SF [MHC‐SF] and Patient‐Reported Outcomes Measurement Information System [PROMIS] domains of anxiety, depression, sleep disturbance, and fatigue).

**Results:**

A total of *N* = 316 LGBTQ+cancer caregivers participated in the survey, and *N* = 14 participated in an interview. Among caregivers, 34.5% reported high financial toxicity (mCOST< 22). The average MHC‐SF score was 38.4 (SD: 16.9, range: 0–70). Mean PROMIS domain T‐scores were: anxiety 59.7 (SD: 10.0, range: 40.3–81.6), depression 56.2 (SD: 10.6, range: 41.0–79.4), sleep disturbance 54.2 (SD: 9.2, range: 32–73.3), and fatigue 54.8 (SD: 11.9, range: 33.7–75.8). In controlled multivariable models, high financial toxicity was significantly associated with poorer mental health (lower MHC‐SF scores [beta = −10.56, 95% CI: −14.47 to −6.65], and higher PROMIS anxiety [beta = 8.54, 95% CI: 6.33–10.74], depression [beta = 7.49, 95% CI: 5.07–9.91], sleep disturbance [beta = 6.33, 95% CI: 4.16–8.50], and fatigue [beta = 8.94, 95% CI: 6.26–11.63]. Qualitative analysis yielded two themes: (1) Financial Stress and Frustration and (2) Negative impacts of financial toxicity on health.

**Conclusion:**

LGBTQ+cancer caregivers experience high financial toxicity that is associated with poor mental health outcomes. Financial toxicity should be addressed in addition to general caregiving‐related stress in this at‐risk population.

## 1 Introduction

Caregivers provide essential, typically unpaid, care to individuals across the cancer care continuum from diagnosis through long‐term survivorship and bereavement [1]. The population of cancer caregivers in the U.S. continues to increase. In 2020, there were an estimated 2.9 million caregivers providing care to an adult with cancer [2]. In 2025, this estimate increased to 4.3 million [3]. Cancer caregiving is a time‐intensive, physically and psychologically demanding experience. Caregivers take on many responsibilities such assisting with activities of daily living, medication management, complex nursing care, and logistical support [1]. The physical and psychological demands of cancer caregiving have far‐reaching impacts on caregivers' mental, physical, and financial health [4, 5, 6].

In addition to the direct care they provide, cancer caregivers may also experience financial toxicity, defined as the objective financial burden and subjective financial distress related to the direct and indirect costs of paying for cancer‐related medical costs and living expenses or impacts on employment and subsequent lost wages [7]. The average cancer caregiver provides an estimated 33 hours of essential care per week, with the majority reporting changes in their employment because of caregiving responsibilities [1]. These employment changes commonly include reducing work hours, taking time off, and retiring early, which can have long‐term financial consequences ranging from reduced income to bankruptcy [8]. Individuals from low‐income households are also at higher risk for cancer caregiving‐related financial toxicity [8]. However, the link between financial toxicity and mental health outcomes has not been sufficiently explored in gender and sexual orientation diverse caregiver populations [9]. Caregiver financial toxicity and poor mental health is also linked to poorer outcomes among the patients they care for, specifically lower treatment adherence and quality of life [10].

Approximately 9.3% of the U.S. population identified as LGBTQ+ in 2025. And national survey data have demonstrated that LGBTQ+people are more likely to be younger and financially vulnerable [11] and more likely to be a primary caregiver than non‐LGBTQ+adults [2, 3]. In the most recent Caregiving in the U.S. 2025 report, LGBTQ+caregivers reported more financial impacts and poorer health than non‐LGBTQ+caregivers [3]. In addition, in a prior report from data used in the present study, we found that LGBTQ+cancer caregivers have substantial financial toxicity related to the costs of cancer care and consistently reported employment changes due to caregiving [12]. In the context of cancer, LGBTQ+cancer survivors are more likely to experience financial toxicity and poor mental health than non‐LGBTQ+cancer survivors [13, 14]. These disparities are driven by anti‐LGBTQ+stigma and resulting societal level conditions such as pre‐existing wealth gaps [11], health care related discrimination and structural barriers to accessing care [15]. LGBTQ+cancer caregivers are likely at similar risk for poor health; however, our understanding of the link between financial toxicity and mental health is limited. Further, the psychological experiences of LGBTQ+cancer caregivers with financial toxicity and impacts on their health have yet to be explored.

To address these gaps in the literature, we conducted a sequential explanatory mixed‐methods study including a cross‐sectional online survey study focused on the financial toxicity and mental well‐being experienced by LGBTQ+cancer caregivers, and follow‐up semi‐structured interviews. This study aimed to assess the relationship between financial toxicity and mental health outcomes among LGBTQ+cancer caregivers and describe perceptions of the link between financial toxicity and mental health.

## 2 Methods

The Understanding the Psychological wellbeing of LGBTQIA+cancer caregivers Impacted by Financial Toxicity (UPLIFT) study was a national sequential explanatory mixed‐methods study that included a cross‐sectional survey of LGBTQ+cancer caregivers in the United States, followed by individual in‐depth interviews with a subset of purposively sampled survey participants. Caregivers were eligible for the UPLIFT study if they were at least 18 years old, provided unpaid care to a cancer patient in the last 3 years, lived in the United States, and identified with at least one LGBTQ+identity. Participant recruitment was conducted through multiple mechanisms, including an academic institution and a market research firm, community and advocacy organizations, and social media advertisements. Survey recruitment began in April 2024 and concluded in December 2024. Participants were compensated $10 for participating in the survey.

Due to the trend of an increasingly high number of fraudulent responses in online surveys [16], a variety of data quality checks were implemented to ensure the authenticity of responses. Responses were excluded if there were duplicate IP addresses, survey completion time of speed less than 10 minutes, and discrepancies between paired open‐ and closed‐ended questions (e.g., sexual orientation and gender). We further checked the quality of our responses using a STATA module that uses IP addresses to identify participants from outside of the US and those using a virtual private network (VPN) to mask their location (participants were asked to turn off VPN use before participating). This study was approved by the Institutional Review Boards of the University of Pittsburgh (STUDY23080142) and University of North Carolina at Chapel Hill (24‐1775).

### 2.1 Survey Design and Measures

The UPLIFT survey was developed by an interdisciplinary team of clinical and non‐clinical cancer health services researchers with expertise in financial toxicity, mental health and psychology, LGBTQ+health, and cancer caregiving. The survey was administered via Qualtrics to facilitate online participation. The survey focused on employment, financial toxicity, psychological experiences (e.g., stigma and mental health outcomes) of LGBTQ+cancer caregivers. Financial toxicity was assessed using a modified version of the 11‐item COmprehensive Score for financial Toxicity (mCOST) [10]. The COST was modified to ensure that the language focused on caregiver experiences rather than patient experiences and to align with the validated measure. The mCOST ranged from 0 to 44 with higher scores indicating less financial toxicity. The mCOST score was dichotomized at a cut‐off score of 22, a commonly used cut‐off for high financial toxicity in the literature (mCOST < 22 = high financial toxicity; mCOST ≥ 22 = low or no financial toxicity) [10].

Measures of mental well‐being included the Mental Health Continuum—Short Form (MHC‐SF) [17], PROMIS anxiety, depression, sleep disturbance, and fatigue [18]. The 14‐item MHC‐SF ranges from 0 to 70, with higher scores indicating better mental well‐being. Anxiety, depression, sleep disturbance, and fatigue were all collected using PROMIS short‐form 4a versions of the concepts, all of which were 4‐item measures. Raw PROMIS scores for each measure were converted to T‐scores per the HealthMeasures guidelines [19]. All PROMIS measures are interpreted such that higher scores indicate greater levels of the concept being measured.

### 2.2 Qualitative Interview Participants and Data Collection

Individuals were recruited for interviews directly from the pool of survey participants who selected that they agreed to being recontacted for a follow‐up interview. Interview recruitment prioritized recruiting LGBTQ+cancer caregivers who reported high financial toxicity as measured by any of the survey's financial toxicity items (mCOST score < 22). Interview recruitment occurred between September and December 2024, overlapping with survey data collection. Interview data collection continued until the study leads (KRT and ARW) deemed the information power to be sufficient. Information power is an alternative to saturation that uses five domains to guide decisions on whether the information collected contains the desired depth and breadth of information: (1) specific study aim, (2) narrow sample, (3) guided by theory, (4) high‐quality dialogue, and (5) a focused analytic strategy [20]. Adequate information power was reached in our study by meeting the criteria outlined in the five domains: (1) interviews focused on specific aim of describing experiences financial toxicity, (2) conducted in a narrow sample of LGBTQ+cancer caregivers, (3) was guided by financial toxicity theories, (4) interviews had high‐quality dialogue, and (5) was analyzed through a focused strategy with multiple cycles of qualitative coding (detailed below) [20]. A more detailed description of the development of the interview guide and an online version of the guide are available elsewhere [12]. Interview participants received additional compensation ($20) for participating in an interview.

### 2.3 Quantitative Data Analysis

Participants who did not complete a full mCOST score were excluded from this analysis, resulting in an analytic sample of *N* = 316 (15 responses excluded). Demographic characteristics were summarized, and Chi‐squared Tests or Fisher's Exact Tests (when small sample size) were used to assess differences in demographic characteristics by high financial toxicity or low/no financial toxicity. Summary statistics were used to generate means, standard deviations (SD), and ranges for each mental health outcome. Two‐way *T*‐tests were then used to assess the difference in each mental well‐being outcome by high financial toxicity or low/no financial toxicity to assess the bivariate association of financial toxicity with the mental well‐being of LGBTQ+cancer caregivers. Multivariable regression was then run for each mental well‐being outcome. Each model controlled for the same variables to maximize comparability and included caregiver age at survey (continuous), race (Black, White, Asian, another or multiple races), ethnicity (non‐Hispanic/Latine, Hispanic/Latine), and caregiving relationship (spouse/significant other/ex‐spouse, sibling, parent/parent‐in‐law, grandparent, friend or chosen family, other relationship). All analyses were complete case and conducted in STATA 17 with a predetermined statistical significance threshold of *p* < 0.05.

### 2.4 Qualitative Data Analysis

Individual interviews were conducted by A.R.W. via Zoom. Interviews were audio‐recorded and transcribed by G.M.R. Transcription. An inductive qualitative analysis was conducted by A.R.W., C.R.M., S.R.J., E.E.K., and K.R.T. [21, 22]. The analytic team, led by A.R.W., first immersed themselves in the data, reading through the transcripts and taking memos on concepts that were repeating or seemed important. The analysis consisted of two cycles of qualitative coding. First open coding was conducted on 6 (43%) of the transcripts by A.R.W., C.R.M., and S.R.J. in Microsoft Word. Open codes were short, coder‐generated descriptive labels that captured the content of that section of text. The open codes were then discussed among the coders and EEK to generate the preliminary coding structure. A.R.W. and CRM then each coded one additional transcript that had not previously been coded (14% of transcripts) into the preliminary codebook in Dedoose. During and after coding the additional two transcripts, A.R.W. and C.R.M. met with the rest of the analytical team to discuss the fit of the coding structure and refine it. During this process, all codes applied to both transcripts were reviewed by A.R.W., C.R.M., and S.R.J. to reach coder consensus on their application. To maximize inter‐coder reliability, coders reached consensus on each code across both transcripts (100% agreement). The remaining transcripts were then coded by A.R.W., code application was double‐checked by C.R.M. Any coder conflicts were resolved during coder meetings. Once all transcripts were coded, A.R.W., C.R.M., E.E.K., and K.R.T. met to synthesize themes. The entire qualitative analysis was grounded in the three domains of financial toxicity, including material, behavioral, and psychological. The psychological financial toxicity‐related content is included in this analysis, while material and behavioral financial toxicity content has been published elsewhere [12]. This study followed the CORE‐Q guidelines.

### 2.5 Data Integration

Data integration occurred throughout study conceptualization, data collection and analysis. First, the quantitative survey and qualitative interview guide were designed in tandem. Second, the qualitative participants were purposively sampled by recruiting individuals who reported high levels of financial hardship. Quantitative and qualitative data were merged based on two concepts (1) financial toxicity and negative emotions and (2) financial toxicity impacts on mental health outcomes in the results and discussion. Joint displays were created to illustrate the connections between quantitative and qualitative results.

## 3 Results

The analytic sample comprised *N* = 316 LGBTQ+cancer caregivers from the UPLIFT study. The average age of caregivers was 41.7 years (SD: 21.2). Caregivers most commonly reported being gay or lesbian (43.8%) or bisexual (46.7%) and cisgender woman (43.5%) and cisgender man (41.2%). The most commonly reported races and ethnicities were White (66.0%), Black (21.4%), and non‐Hispanic/Latine (89.3%). Most caregivers cared for a parent/parent‐in‐law (38.9%) or spouse/partner (16.5%; Table 1). Over one third of caregivers reported high financial toxicity—COST score less than or equal to 22 (34.5%, *n* = 109). Some caregiver demographics were significantly different by high financial toxicity and low/no financial toxicity, including sexual orientation, gender, race, and caregiver relationship (Table 1). The mean MHC‐SF score was 38.4 (SD: 16.9, range: 0–70), PROMIS anxiety T‐score was 59.7 (SD: 10.0, range: 40.3–81.6), PROMIS depression score was 56.2 (SD: 10.6, range: 41.0–79.4), PROMIS sleep disturbance score was 54.2 (SD: 9.2, range: 32–73.3), and PROMIS fatigue score was 54.8 (SD: 11.9, range: 33.7–75.8). Qualitative themes included: (1) Financial Stress and Frustration, and (2) Negative Impacts on Health. See Figure 1 for illustrative quotes.

**TABLE 1:** Demographic characteristics of LGBTQ+cancer caregivers by high and low/no financial toxicity.

	Overall sample (*N* = 316)		High financial toxicity (*N* = 109)		Low/no financial toxicity (*N* = 207)		*p*‐values	Interview sample (*N* = 14)	
	Mean	SD	Mean	SD	Mean	SD		Mean	SD
Age at survey^a^	41.7	21.2	42.3	1.3	41.3	24.2	0.7	50.1	13.6
	***N***	**%**	***N***	**%**	***N***	**%**			
Sexual orientation^b^							0.02		
Gay or lesbian	138	43.8	54	50.0	84	40.6		9	64.3
Bisexual	147	46.7	43	39.8	104	50.2		4	28.6
Asexual	13	4.1	3	2.8	10	4.8		—	—
Other orientation	17	5.4	8	7.4	9	4.4		1	7.1
Gender^a^							< 0.001		
Cisgender man	129	41.2	51	48.1	78	37.7		7	50.0
Cisgender woman	136	43.5	31	29.3	105	50.7		3	21.4
Transgender man	9	2.9	4	3.8	5	2.4		—	—
Transgender woman	11	3.5	5	4.7	6	2.9		1	7.1
Nonbinary	17	5.4	6	5.7	11	5.3		2	14.3
Another or multiple gender	11	3.5	9	8.5	2	1.0		1	7.1
Race^c^							0.014		
Black	66	21.4	20	18.9	46	22.7		4	28.6
White	204	66.0	68	64.2	136	67.0		9	64.3
Asian	13	4.2	2	1.9	11	5.4		—	—
Another or multiple races	26	8.4	16	15.1	10	4.9		1	7.1
Ethnicity^c^							0.8		
Non‐hispanic/latine	276	89.3	93	88.6	183	89.7		12	92.3
Hispanic/latine	33	10.7	12	11.4	21	10.3		1	7.7
Education							0.8		
High school/GED or less	51	16.1	14	12.8	37	17.9		1	7.7
Some college, no degree	78	24.7	29	26.6	49	23.7		2	15.4
Associate's degree	57	18.0	19	17.4	38	18.4		—	—
Bachelor's degree	99	31.3	37	33.9	62	30.0		7	53.8
Master's or advanced degree	31	9.8	10	9.2	21	10.1		3	23.1
Rurality^d^							0.2		
Rural (RUCA 4–10)	36	11.7	16	14.7	20	10.1		2	14.3
Non‐rural (RUCA 0–3)	271	88.3	93	85.3	178	89.9		12	85.7
Caregiver relationship							0.02		
Spouse/significant other/ex‐spouse	52	16.5	24	22.0	28	13.5		3	21.4
Sibling	18	5.7	3	2.8	15	7.3		—	—
Parent/parent‐in‐law	123	38.9	45	41.3	78	37.7		4	28.6
Grandparent	42	13.3	9	8.3	33	15.9		2	14.3
Friend or chosen family	45	14.2	11	10.1	34	16.4		3	21.4
Other relationship	36	11.4	17	15.6	19	9.2		2	14.3

*Note:* Comparisons were made using Chi‐squared Tests or Fisher Exact Tests when appropriate.

^a^ Missing 3.

^b^ Missing 1.

^c^ Missing 7.

^d^ Missing 9.

**FIGURE 1:**
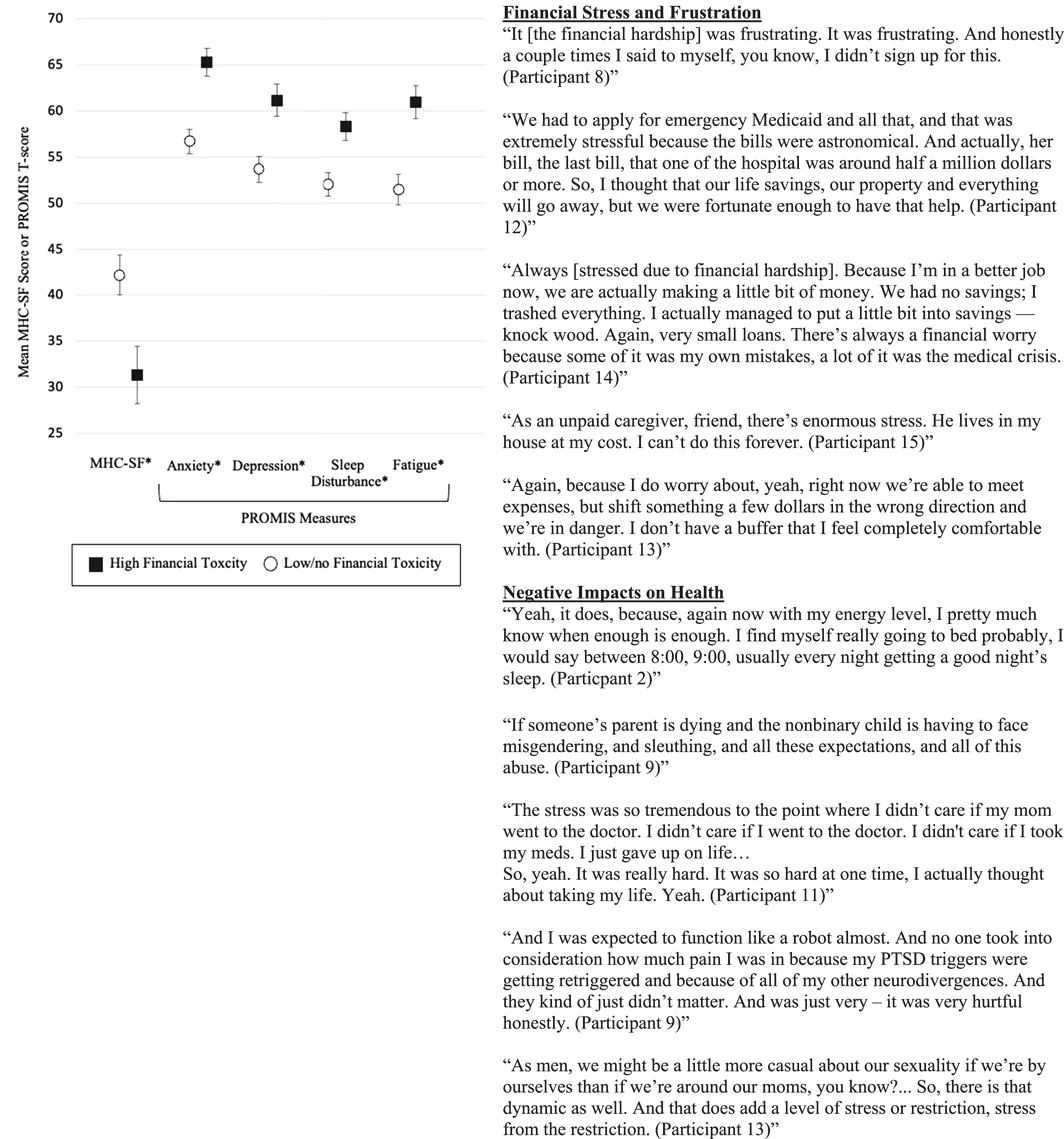
Associations of financial toxicity with health outcomes and qualitative themes and illustrative quotes. MHC‐SF: Mental Health Continuum Short Form. Two‐way T‐tests were used to assess difference. Sample size varied across measures due to missing data; total sample sizes used in testing were *n* = 316 for MHC‐SF, *n* = 313 for anxiety, n = 314 for depression, *n* = 307 for sleep disturbance and fatigue.

The first qualitative theme, Financial Stress and Frustration, was derived from participants' descriptions of their reactions to the costs of providing care. Among nearly all caregivers, the discussion of caregiving costs was accompanied by an assessment of its stressfulness and frustration. Caregivers often reported that their stress stemmed from the all‐consuming nature of caregiving, which reduced their long‐term financial security, including by depleting savings and incurring debt. One caregiver shared their frustration by stating: *[The financial stress was] Frustrating, I would use a stronger word than that. But I'm not going to. It was unfortunate. I don't know what else I could have done. Sometimes I feel the blame or take the blame because maybe there was a keyhole that I missed that I didn't unlock. It was just disappointing.* (Participant 5).

Aligning with qualitative findings, in bivariate analyses, high financial toxicity was significantly associated (*p* < 0.001) with indicators of poor mental health including lower MHC‐SF scores (MHC‐SF: 42.2, 95% CI: 40.0–44.4 vs. 31.3, 95% CI: 28.2–34.4) and higher PROMIS anxiety (65.3, 95% CI: 63.8–66.8 vs. 56.7, 95% CI: 55.4–58.1), depression (61.2, 95% CI: 59.4–62.9 vs. 53.7, 95%CI: 52.3–55.1), sleep disturbance (58.4, 95% CI: 56.8–59.8 vs. 52.0, 95% CI: 50.8–53.3) and fatigue (60.9, 95% CI: 59.2–62.7 vs. 51.5, 95% CI: 49.9–53.1; Figure 1). In multivariable models controlling for age, race, ethnicity, and caregiving relationship, high financial toxicity was significantly associated with all health outcomes (Table 2). High financial toxicity was negatively associated with mental well‐being (MHC‐SF; beta = −10.56, 95% CI: −14.47 to −6.65) and positively associated with PROMIS anxiety (beta = 8.54, 95% CI: 6.33–10.74), depression (beta = 7.49, 95% CI: 5.07–9.91), sleep disturbance (beta = 6.33, 95% CI: 4.16–8.50), and fatigue (beta = 8.94, 95% CI: 6.26–11.63) in comparison to caregivers with low or no financial toxicity.

**TABLE 2:** Multivariable linear regression models of the association of financial toxicity and PROMIS health related quality of life measures among LGBTQ+cancer caregivers.

	High financial toxicity *N* = 109	Low financial toxicity *N* = 207	Unadjusted models		Adjusted models	
	Mean	Mean	Beta	95% CI	Beta	95% CI
MHC‐SF	31.32	42.19	−10.87	−14.63–−7.11	−10.56	−14.47–−6.65
PROMIS anxiety	65.31	56.71	8.61	6.46–10.76	8.54	6.33–10.74
PROMIS depression	61.18	53.67	7.51	5.17–9.84	7.49	5.07–9.91
PROMIS sleep disturbance	58.36	52.02	6.34	4.27–8.40	6.33	4.16–8.50
PROMIS fatigue	60.95	51.50	9.45	6.86–12.04	8.94	6.26–11.63

*Note:* Multivariable models control for age, race, ethnicity, and caregiving relationship. Sample size varied across measures due to missing data; total sample sizes used in unadjusted models were *n* = 316 for MHC‐SF, *n* = 313 for anxiety, *n* = 314 for depression, *n* = 307 for sleep disturbance and fatigue.

The qualitative theme, Negative Impacts on Health, further reflected the quantitative findings. Caregivers commonly discussed financial toxicity in the context of their overall mental and physical health. Many caregivers identified how the financial toxicity affected their mental health but preferred to discuss it more holistically, such that the toxicity is not experienced independently of other caregiving stressors and does not affect only one aspect of their health. For example, one caregiver stated, *All of a sudden someone in my family is sick. And you don't have as much money in your pocket, and you're not working as much, and then you're expected to be there for someone and stuff, which I was glad to do. But—yeah. It does—it's totally connected to anxiety* (Participant 6), while another caregiver stated, *I could barely take care of myself. I could barely exercise or make healthy meals. I had to cancel all of my doctor's appointments because I couldn't do it. I just didn't have time. I didn't have time to take sick time where I didn't have it.* (Participant 9).

## 4 Discussion

In our U.S.‐based explanatory sequential mixed‐methods study, we found that among LGBTQ+cancer caregivers, higher cancer related‐financial toxicity was consistently associated with worse mental health outcomes. Financial toxicity is a common experience among cancer caregivers, and our prevalence findings are consistent with national estimates. In our sample, 34.5% reported high financial toxicity, which is higher than national prevalence estimates (with majority non‐LGBTQ+populations) of 25% among cancer caregivers [1] and 20% among all caregivers [3]. Higher financial toxicity in our LGBTQ+sample is consistent with the 2025 Caregiving in the U.S. report finding that LGBT caregivers report more financial impacts and greater financial toxicity than non‐LGBT caregivers [3]. Furthermore, a previously published UPLIFT analysis sets the stage for this analysis by describing frequent experiences of employment changes, financial hardship, and stigma among LGBTQ+cancer caregivers [12]. The findings of this analysis highlight not only the prevalence of financial toxicity but also the association of financial toxicity with mental health outcomes among LGBTQ+cancer caregivers, an underrepresented group in research despite the growing population.

Cancer caregivers commonly experience caregiving‐related stress and downstream effects on health outcomes [23]. Financial toxicity is one component of caregiving‐related stress that may worsen their health outcomes. In our study, we found that experiences of high financial toxicity are associated with mental health effects, including worse anxiety, depression, sleep disturbance, and fatigue, than those of individuals with low financial toxicity. The study findings add to our understanding of caregiving‐related stress by demonstrating the specific effects of financial toxicity on mental health outcomes. Our findings corroborate that the association between caregiving‐related stress and health outcomes is highly complex [24] and prevalent in LGBTQ+cancer caregiving populations. Qualitative evidence demonstrates that caregivers experience financial impacts due to the direct costs of medical treatment overwhelming the financial resources of the patient and caregiver, and when caregiving demands require time and effort in conflict with workplace hours [25]. Financial impacts related to reducing workplace hours to provide care may be further exacerbated by workplace disparities such as a lack of protections and eligibility for workplace benefits for LGBTQ+individuals if they are not blood family or spouses, leading to greater financial impacts when LGBTQ+caregivers need to provide care.

In the context of cancer caregiving, most interventions focus on addressing stress coping and social support, with little attention given to addressing financial stressors [26]. Although most cancer caregivers experience financial impacts [3], LGBTQ+cancer caregivers report more financial impacts and greater financial toxicity [2, 3]. LGBTQ+cancer caregivers likely require additional intervention focused on addressing financial impacts and experienced financial toxicity in addition to general stress and coping interventions. Further, existing financial toxicity interventions typically focus on only the patient or spousal and blood family caregivers [27]. LGBTQ+cancer caregivers have unique needs related to financial toxicity. Additional interventions need to be developed that are specific to their experiences, as they may not be eligible or able to utilize financial solutions that are used by spouses and blood family.

### 4.1 Clinical Implications

In clinical settings, individuals working with cancer caregivers should take into consideration the direct and indirect impacts of the costs of caregiving for an individual with cancer, particularly for caregivers who are LGBTQ+. Documentation, screening and support for individuals with cancer experiencing financial toxicity varies widely in cancer care settings [28], and even fewer document, screen, and support caregivers experiencing financial toxicity [29]. Supportive cancer care programs should include caregivers by documenting them, screening them for financial toxicity, and addressing immediate basic needs (e.g., housing and food). Because LGBTQ+individuals are more likely to face workplace discrimination [3], be relied on as caregivers [3], and have fewer financial reserves and resources they are at even greater risk for financial toxicity. Despite being at greater risk for financial toxicity, LGBTQ+cancer caregivers may not be eligible for the same financial supports as blood family or spousal cancer caregivers. Individuals providing financial navigation or assistance with applying for financial help should take into consideration eligibility and applicability of recommended resources and solutions.

### 4.2 Study Limitations

Several limitations should be considered when interpreting the study's findings. First, this is a cross‐sectional study, therefore, the direction of the relationships between financial toxicity and mental health outcomes cannot be determined. Second, due to its cross‐sectional design and use of retrospective surveys, the study's findings rely on caregiver recall, which may be subject to recall bias or reporting errors. Data were not collected on specific financial costs associated with cancer caregiving; instead, they focused on perceived impacts of cancer caregiving. To address these limitations, future studies are needed to better understand LGBTQ+caregiver health disparities in other diseases, and longitudinal data are needed to further evaluate the temporality between financial toxicity and effects on mental health outcomes and duration of effects on mental health. Sexual orientation and gender identity data are needed in future research to continue developing our understanding of at‐risk populations.

## 5 Conclusions

In this mixed‐methods study of U.S.‐based LGBTQ+cancer caregivers, financial toxicity was consistently strongly associated with mental health outcomes. Increasing costs of cancer, and reliance on caregivers for cancer treatment and management will continue to increase overall caregiving toxicity; the financial impacts must be assessed and addressed in addition to the overall caregiving toxicity. Specific tailored content is needed in financial interventions for individuals who are LGBTQ+or providing care to someone who is not blood family or spousal, as their needs and protections differ from spousal and blood family caregivers. Future changes to policy are also necessary to reduce LGBTQ+cancer caregiver financial toxicity. These changes include adding inclusive language to policies that expand eligibility for benefits to non‐spousal caregivers and improve workplace protections for LGBTQ+caregiving populations.

## Funding

This project was supported by an Agency for Healthcare Research and Quality dissertation research award R36HS030175 (PI: Waters), a pilot award (PI: Tan) through the Emotional well‐being and economic burden research network funded by the National Center for Complementary and Integrative Health (NCCIH), the Office of Behavior and Social Sciences Research (OBSSR), the Office of Disease Prevention and National Institutes of Health Office of the Director under award number U24AT011310, and S. Jones was supported as a pre‐doctoral trainee under the Cancer Control Education Program at the University of North Carolina at Chapel Hill funded by the National Cancer Institute of the National Institutes of Health under award number T32CA057726. The content is solely the responsibility of the authors and does not necessarily represent the official views of the National Institutes of Health.

## Conflicts of Interest

The authors declare no conflicts of interest.

## Data Availability

The data that support the findings of this study are available on request from the corresponding author. The data are not publicly available due to privacy or ethical restrictions.
